# Spatio-Temporal Differences in Nitrogen Reduction Rates under Biotic and Abiotic Processes in River Water of the Taihu Basin, China

**DOI:** 10.3390/ijerph15112568

**Published:** 2018-11-16

**Authors:** Jiaxun Guo, Lachun Wang, Xiya Guo, Gengmao Zhao, Jiancai Deng, Chunfen Zeng

**Affiliations:** 1 School of Geographic and Oceanographic Sciences, Nanjing University, Nanjing 210023, China; DG1727009@smail.nju.edu.cn (J.G.); wang6312@nju.edu.cn (L.W.); zengchunfen@hotmail.com (C.Z.); 2 Nanjing Institute of Geography and Limnology, Chinese Academy of Sciences, Nanjing 210008, China; Everlasting_west@163.com; 3 College of Resources and Environmental Sciences, Nanjing Agricultural University, Nanjing 210095, China; seawater@njau.edu.cn

**Keywords:** nitrogen reduce, spatio-temporal differences, biotic and abiotic, in-situ, Taihu Basin

## Abstract

Understanding spatio-temporal differences in nitrogen (N) transformation, transport and reduction rates in water bodies is critical to achieve effective mitigation of river eutrophication. We performed culture experiments in six rivers in the Taihu Basin using a custom made in-situ experimental apparatus. We investigated spatio-temporal differences in reduce processes and rates of different N forms and assessed the contribution of biological processes to dissolved inorganic N (DIN) reduce. Results showed that biological processes played a major role in N reduction in summer, while non-microbial processes were dominant in winter. We observed significant spatial and temporal differences in the studied mechanisms, with reduction rates of different N compounds being significantly higher in summer and autumn than spring and winter. Reduction rates ranged from 105.4 ± 25.3 to 1458.8 ± 98.4 mg·(m^3^·d)^−1^ for total N, 33.1 ± 12.3 to 440.9 ± 33.1 mg·(m^3^·d)^−1^ for ammonium, 56.3 ± 22.7 to 332.1 ± 61.9 mg·(m^3^·d)^−1^ for nitrate and 0.4 ± 0.3 to 31.8 ± 9.0 mg·(m^3^·d)^−1^ for nitrite across four seasons. Mean DIN reduction rates with and without microbial activity were 96.0 ± 46.4 mg·(m^3^·d)^−1^ and 288.1 ± 67.8 mg·(m^3^·d)^−1^, respectively, with microbial activity rates accounting for 29.7% of the DIN load and 2.2% of the N load. Results of correlation and principal component analysis showed that the main factors influencing N processing were the concentrations of different N forms and multiple environmental factors in spring, N concentrations, DO and pH in summer, N concentrations and water velocity in autumn and N concentrations in winter.

## 1. Introduction

Nitrogen (N) is an essential nutrient element for all organisms, but excessive inputs of bioavailable N from anthropogenic activities have severely impacted aquatic ecosystems [1,2,3]. Excess amounts of N can cause eutrophication and deterioration of ecosystems and water quality. For example, two thirds of the coastal rivers and bays in the United States are moderately to severely degraded due to N pollution (U.S. Environmental Protection Agency, 2001), and more than 85% of the lakes and about 82% of the 532 major rivers in China experienced eutrophication and water quality deterioration due to heavy N pollution [4]. Processes such as organic matter burial in sediment, denitrification, sediment adsorption and plant and microbial uptake can remove N transported in rivers and streams, thereby reducing the amount of N exported to lakes and reservoirs [5]. Therefore, reduction of N pollution in surface waters should not only focus on decreasing N inputs but also on improving N removal capacity in water bodies [6,7]. Measures commonly adopted to control N pollution include reducing pollutants from industrial and domestic sources and intercepting waterborne pollutants before they enter rivers. Examples of such measures are the “Zero Pollution” actions in the Lake Taihu region in China [7,8].

When point source pollutants discharged directly into water bodies are controlled effectively, rivers are the main sources of pollutants to lakes and oceans. Aquatic ecosystems naturally possess self-purification capacities. After N enters aquatic ecosystems, part of it is removed through biological processes (e.g., biodegradation and adsorption) and abiotic processes (e.g., dilution, deposition, volatilization and oxidation [9]. In aquatic ecosystems, bacterial denitrification is considered the primary pathway for permanent N removal via N_2_ production, with other processes acting as only temporary N storage mechanisms [10]. Bacterial denitrification and N removal rates have been estimated in several rivers and lakes. For example, denitrification rates were 0.25–0.63 mmol N·L^−1^·d^−1^ in Poyang Lake’s suspended sediments, 0–56.4 mg N·m^−2^·d^−1^ in sediments in the Taihu Basin and 274–2111 mg N·m^−2^·d^−1^ in the water of a Lake Superior estuary; N_2_O emission rates were 0.04–56.47 mg N·m^−2^·d^−1^ in urban rivers and 0.01–80.9 mg N·m^−2^·d^−1^ in runoff rivers; N_2_ production rates ranged between 19.08 and 375.44 mg N_2_-N·m^−2^·d^−1^ in western Lake Taihu [11,12,13,14,15]. Processes contributing to N removal in water and sediments are affected by several environmental factors, including water temperature and concentrations of NO_3_^−^-N, dissolved oxygen (DO) and suspended solids [13,14,15,16].

Most studies have focused on N removal rates out of water. Although permanent N removal is achieved by microbial processes, sedimentation of suspended solids containing N adsorbed to their surface can also contribute to N reduce. Previous studies have generally focused on biological mechanisms, while the contribution of other processes to N reduction rates has not been studied extensively. Furthermore, a large proportion of studies on N reduction was conducted in the laboratory using simulations that are often unable to accurately capture complex environmental processes. To improve the current understanding of N reduction rates in natural rivers, we investigated N reduction rates in six rivers in the Taihu Basin using a custom made in-situ experimental apparatus. In each river, we analyzed the N concentrations of different treatments in the culture bottles before and after the experiment. Based on the results, we quantified N reduction rates and evaluated the main processes contributing to N reduce. Specifically, we assessed the relative contribution of biotic and abiotic processes and explored the effects of different environmental factors on N reduce processes. Our study is based on two hypotheses, (1) the biochemical activity can significantly reduce N in natural water within one day, and (2) the device can attain the same environmental conditions found in each river.

## 2. Materials and Methods

### 2.1. Site Description

The Lake Taihu Basin is located in the center of the Yangtze River Delta in southeastern China, one of China’s most economically developed regions. The region is characterized by a dense river network and is heavily populated, with a large portion of the population residing along rivers and leading to developed industry and intensive agriculture. The region is dominated by a subtropical monsoon climate, with a rainy season (average amount of rainfall: 750–850 mm) occurring from May to September and alternating with a dry season (average rainfall: 250–350 mm). Annual mean air temperature varies between 14.9 °C and 16.2 °C.

**Figure 1:**
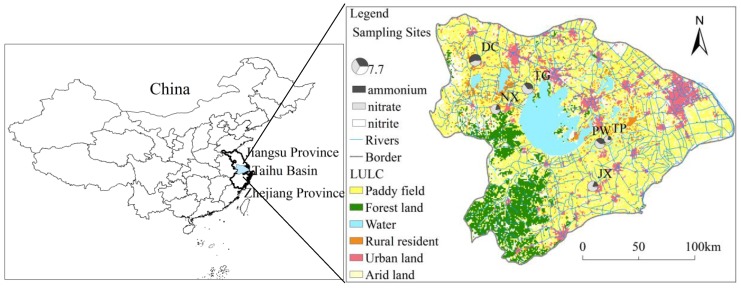
Sampling sites and Land Use/Land Cover (LULC) in the Taihu Basin. The LULC data set was provided by the Data Center for Resources and Environmental Sciences, Chinese Academy of Sciences (RESDC) (http://www.resdc.cn).

We sampled six rivers in the Lake Taihu region (Figure 1) in July 2015 and February, October and December 2016. Sampling months were selected to represent summer, spring, autumn and winter conditions, respectively. Physico-chemical parameters measured at each sampling site in spring, summer, autumn and winter were showed in Table 1. The sampling points were conducted in six rivers including the Danjinlicao River (point DC), Beijing-Hangzhou Canal-Jiaxin (point JX), Beijing-Hangzhou Canal-Pingwang (point PW), Nanxi-Xijiu River (point NX), Taige River (point TG), and Taipu River (point TP), which span a diverse range of environmental conditions, functions and geographic features. The main sources of N pollution to these rivers include industrial wastewater, domestic sewage and agricultural fertilizers from the surrounding areas. Specifically, DC is located in the city of Jintan and is mainly used for shipping, with an average dissolved inorganic N (DIN) concentrations of 4.29 mg·L^−1^ (56.68% as NH_4_^+^-N and 41.07% as NO_3_^−^-N). Similarly, JX is also mainly used for shipping and N pollution to this river derives from sewage and industrial wastewater, resulting in average DIN concentrations of 3.48 mg·L^−1^ (18.84% as NH_4_^+^-N and 77.73% as NO_3_^−^-N). NX is located in the city of Yixing, where it supports agricultural activities and shipping, and it exhibits average DIN concentrations of 3.01 mg·L^−1^ (20.08% as NH_4_^+^-N and 76.32% as NO_3_^−^-N). TG is located in the town of Fenshui, the main N sources to this river are domestic sewage and industrial wastewater and the average DIN concentrations are 4.01 mg·L^−1^ (35.20% as NH_4_^+^-N and 60.98% as NO_3_^−^-N). PW and TP are located in the Wujiang District, Suzhou, and their average DIN concentrations amount to 3.44 mg·L^−1^ (38.73% as NH_4_^+^-N and 58.54% as NO_3_^−^-N) and 1.93 mg·L^−1^ (21.02% as NH_4_^+^-N and 76.59% as NO_3_^−^-N), respectively. Both sampling sites are located in the main channel, but PW is surrounded by an urban area, while TP is located in a predominantly rural area.

### 2.2. Experimental Set-Up and Analyses

**Figure 2:**
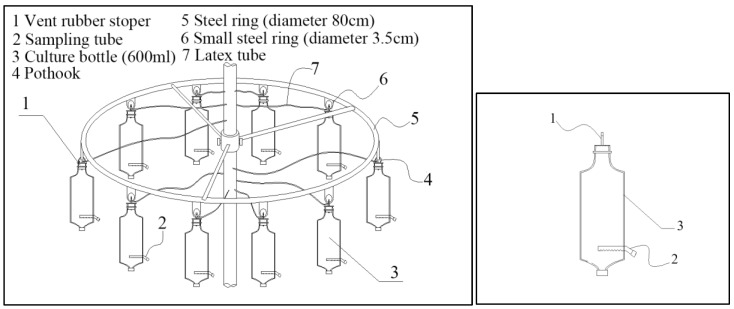
Diagram of the experimental apparatus used in this study.

We developed an experimental apparatus (Patent No. ZL201520816420.7) to measure in-situ N reduction rates (Figure 2). The device is made of a steel ring with a diameter of 80 cm and height of 2.5 cm. The steel ring is connected to a central axle by three 2 cm-wide steel plates. Twelve small steel rings with a diameter of ca. 3.5 cm each was welded along the ring at regular intervals, each carrying a 600-mL culture bottle and a temperature probe. To ensure aerobic conditions in the culture bottles throughout the duration of the experiment, the inside of the culture bottles was connected to the atmosphere through a latex tube. To attain the same environmental conditions (e.g., water temperature and water disturbance) found in each river, the experimental apparatus was submerged under river water at a depth of 50 cm.

We set up and performed an experiment in each river to measure N reduction rates and investigate the effects of environmental factors (DO, pH, SS, velocity and water flow) on N reduction rates under in-situ riverine conditions. The experiment consisted of two treatments, a control and a microbial inhibition treatment, the latter consisting in the addition of 2 mL 0.5% HgCl_2_. For the controls, which reflected N reduce under typical riverine conditions, three culture bottles were filled with river water collected near both river shores and from the open channel at a depth of 50 cm. For the inhibition treatments, three culture bottles were filled with water and added with 2 mL 0.5% HgCl_2_, thereby reflecting N reduce in river water without microbial activity. The water bottles were sealed with a rubber stopper to prevent air exchanges through the bottle top and hanged in the apparatus (Figure 2), and then were placed in the river water.

To avoid disturbing suspended solids settled at the bottom of the bottles, the experimental apparatus was taken out of the water slowly after reaching the preset incubation time (1 day) and then water samples were immediately collected from the culture bottles in the field. Samples of culture water from the upper layer of each bottle were collected from a side sampling port, while samples of water and suspended solids from the bottom of each bottle were collected from a bottom sampling port. The volume of the upper and lower parts of water was measured with a measuring cylinder after being transported back to the laboratory. Water samples were placed in ice for transport to the laboratory, filtered (Whatman GF/C filters, 0.45 μm pore size) and frozen until analysis.

Total N (TN), NH_4_^+^-N, NO_3_^−^-N, NO_2_^−^-N and a set of physico-chemical parameters were measured in each river in spring, summer, autumn and winter. The original water was analyzed for TN using potassium persulfate oxidation with detection limits of 0.001 mg·L^−1^, and suspended solids were measured by weight method with detection limits of 0.0001 mg·L^−1^. Filtered water was analyzed for NH_4_^+^-N, NO_3_^−^-N and NO_2_^−^-N using a flow injection analyzer with detection limits of 0.1, 0.01 and 0.01 mg·L^−1^, respectively (Skalar Analytical, Breda, The Netherlands). The DIN was calculated by summing the NH_4_^+^-N, NO_3_^−^-N and NO_2_^−^-N. At each sampling site, a multi-parameter water quality probe (YSI Inc., Yellow Springs, OH, USA) was used to measure water temperature, DO and pH, and the River Surveyor M9 (SonTek, Xylem Inc., Rye Brook, NY, USA) was used to measure water velocity and flow across each river’s cross-section. The water temperature, DO and pH were measured only once at the beginning of the experiment, and the water velocity and flow were measured at the beginning and the end of the experiment.

### 2.3. Statistical Analyses

The rate of change in N concentration (*R_c_*) in the upper and bottom layer of each bottle was calculated using Equation (1). In the absence of microbial activity, a theoretical rate of change can be calculated for the bottom layer ($T_{R_{2}}$) using Equation (2), which assumes that the N reduction in the upper bottle layer is transferred to the bottom layer. The observed rate of change in bottom water N concentration (*R*_2_) was calculated using Equation (3). The absolute N reductions rates (*R_a_*) was calculated using Equation (4), which quantifies the total reduction rates of different N forms.

(1) $$R_{c}=\frac{C_{0}-C_{x}}{C_{0}}\times 100$$

(2) $$T_{R_{2}}=\frac{C_{0}-C_{1}}{C_{0}}\times \frac{V_{1}}{V_{2}}$$

(3) $$R_{2}=\frac{C_{0}-C_{2}}{C_{0}}$$

(4) $$R_{a}=\frac{C_{0}-C_{0}^{\prime }}{t}\times 1000$$

In the above equations, *C*_0_ is the initial N concentrations, $C_{0}^{\prime }$ is the N concentrations after incubation, t is the incubation time, *C_x_* is the N concentrations after incubation in the upper (*x* = 1, *C*_1_) and bottom (*x* = 2, *C*_2_) layer of the culture bottle and *V*_1_ and *V*_2_ are the upper and bottom sampled water volume.

Data were checked for normality and homogeneity of variance prior to statistical analyses. Spatial and temporal differences in mean reduction rates among sites and seasons were tested with one-way analysis of variance (ANOVA) followed by Fisher’s Least Significant Difference (LSD) test. Relationships between reduction rates and measured environmental variables were examined with correlation and principal component analysis (PCA). All statistical analyses were performed in SPSS version 19.0 (SPSS Inc., Chicago, IL, USA).

## 3. Results

### 3.1. Reduce Processes of Different N Forms

**Figure 3:**
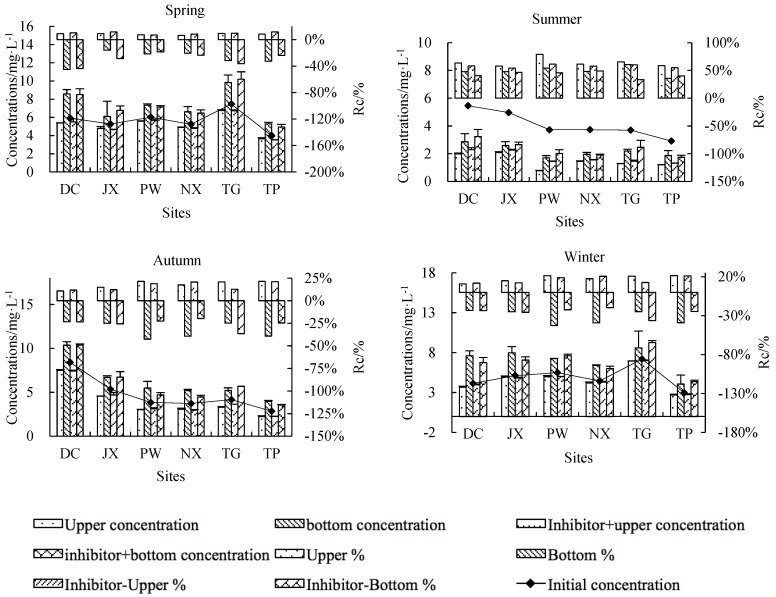
Initial TN (total N) concentrations (line), final upper- and bottom-layer TN concentrations (lower histogram) and percent change from initial TN concentrations (upper histogram). A negative percentage indicates an increase in concentrations and vice versa.

Concentrations of TN in the upper and bottom layers changed significantly after incubation (Figure 3). Compared to initial TN concentrations, upper TN concentrations decreased and bottom concentrations increased in spring, autumn and winter, while both upper and bottom TN concentrations decreased in summer compared to initial TN concentrations. This general pattern was observed across all treatments and sites, although rates of change differed among treatments, sites and seasons. Percentage changes in TN concentrations were 6.41–79.19% for control upper water, −44.94–60.47% for control bottom water, 7.02–61.26% for inhibitor-treated upper water and −43.92–48.93% for inhibitor-treated bottom water. TN reduced from the upper layer was transferred to the bottom layer in both treatments in spring, autumn and winter, suggesting that sedimentation of suspended solids was the primary TN reduce process in these seasons. Summer results suggested that microbial activity played an important role in TN reduction.

**Figure 4:**
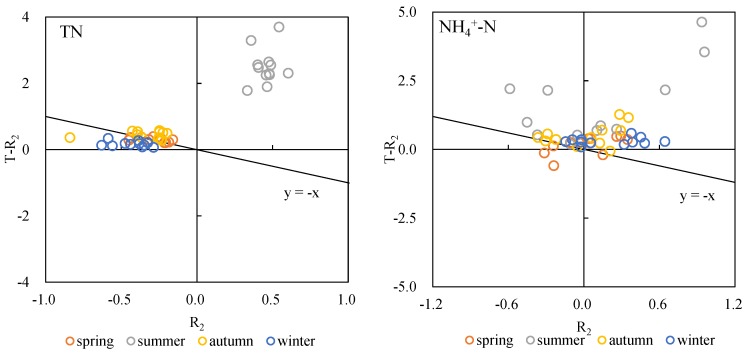

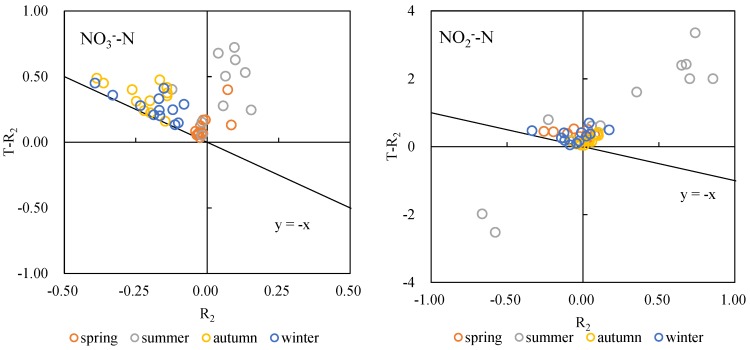
Scatterplot of T-R_2_ (theoretical rate of change of bottom N concentrations, *y*-axis) vs R_2_ (experimentally measured rate of change of bottom N concentrations, *x*-axis). Points lying on the y = −x line exhibit upper concentrations loss rates equaling bottom concentrations increase rates as a result of physical deposition in absence of microbial activity.

A scatterplot of the theoretical vs. measured rate of change for the bottom layer shows that most spring, autumn and winter data points are distributed in quadrant II, close to the y = −x line, while summer data points are located in quadrant I (Figure 4). These results indicate that sedimentation was the main TN reduction process in spring, autumn and winter, while microbial activity contributed significantly in summer.

**Figure 5:**
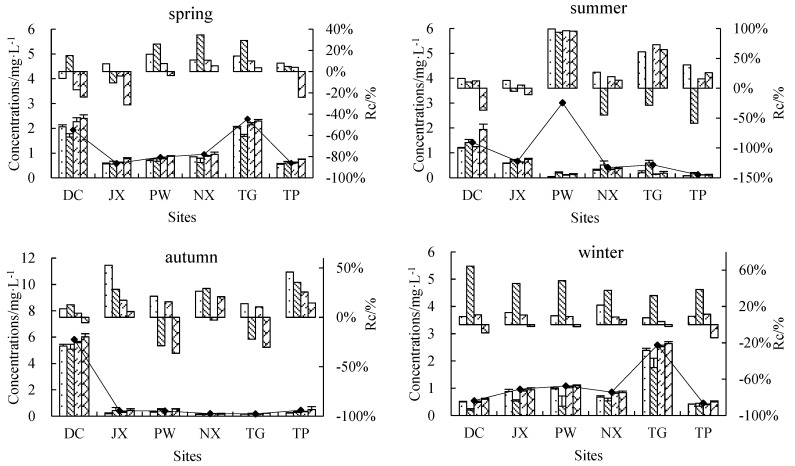

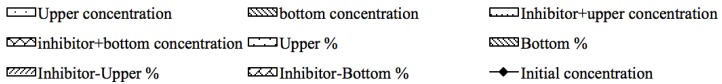
Initial NH_4_^+^-N concentrations (line), final upper- and bottom-layer NH_4_^+^-N concentrations (lower histogram) and percent change from initial NH_4_^+^-N concentration (upper histogram). A negative percentage indicates an increase in concentrations and vice versa.

Rates of change in upper and bottom NH_4_^+^-N concentrations showed significant differences among seasons and sampling sites, although no clear seasonal and spatial pattern emerged (Figure 5). In general, NH_4_^+^-N concentrations decreased in most sites, especially in PW, which showed an average percentage decrease of 96.25%. In some sites, such as DC, PW and TP, a decrease in bottom NH_4_^+^-N was observed in inhibitor-treated bottles, suggesting the occurrence of chemical N reduce processes other than biological activity. Controls showed higher NH_4_^+^-N rates of change than inhibition treatments, illustrating the role of microorganisms in processing N in natural water bodies.

Almost all data points representing the relationship between theoretical and observed rate of change in bottom NH_4_^+^-N concentration lie in quadrant I (25 points) and II (19 points), with only two points appearing in each of quadrant III and IV, respectively (Figure 4). Most points in quadrant II lie above the y = −x line, suggesting the occurrence of biochemical reduce of NH_4_^+^-N in natural water.

**Figure 6:**
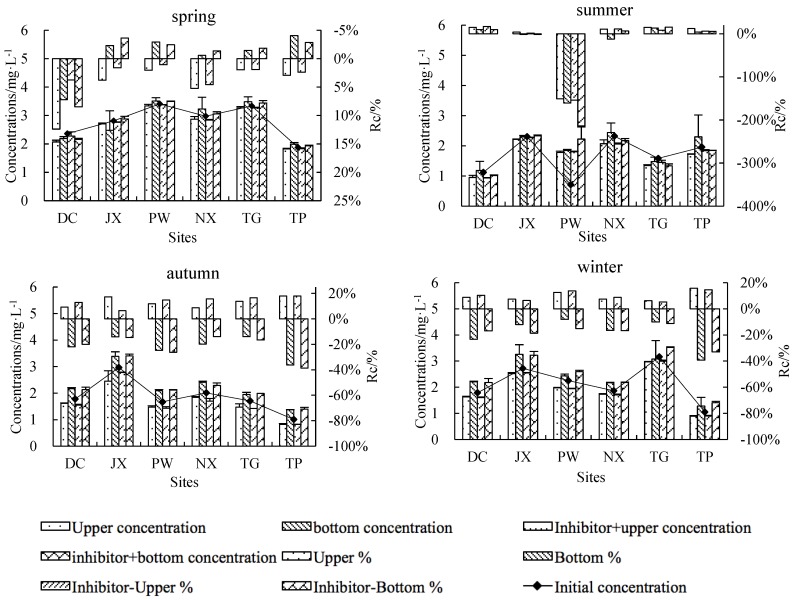
Initial NO_3_^−^-N concentrations (line), final upper- and bottom-layer NO_3_^−^-N concentrations (lower histogram) and percent change from initial NO_3_^−^-N concentrations (upper histogram). A negative percentage indicates an increase in concentrations and vice versa.

Upper NO_3_^−^-N concentrations decreased while bottom NO_3_^−^-N concentrations increased at all sites in spring (except DC), autumn and winter (Figure 6). At all sites rates of change were higher in autumn and winter than in spring (1.95–12.39% for upper control water, −4.06–7.21% for bottom control water, 1.06–4.55% for inhibitor-treated upper water and −3.63–8.48% for inhibitor-treated bottom water). All sites had the lowest rates of change in summer (−12.28–16.93%) with the exception of PW, which showed an average summer rate of change of −169.75%. Percentage decreases were higher in upper control water than in inhibitor-treated upper water, suggesting that biological activity played a key role in the NO_3_^−^-N reduce process relative to physical deposition.

In the plot of theoretical vs. observed bottom rates of change, most summer data points lie in quadrant I (Figure 4), indicating significant biological activity. Two data points belonging to PW and not shown in Figure 4 are located in quadrant III (coordinates [1.6, 7.0] and [2.1, 5.7]). The remaining points are located in quadrant II and above the y = −x line, illustrating the influence of biochemical activity.

**Figure 7:**
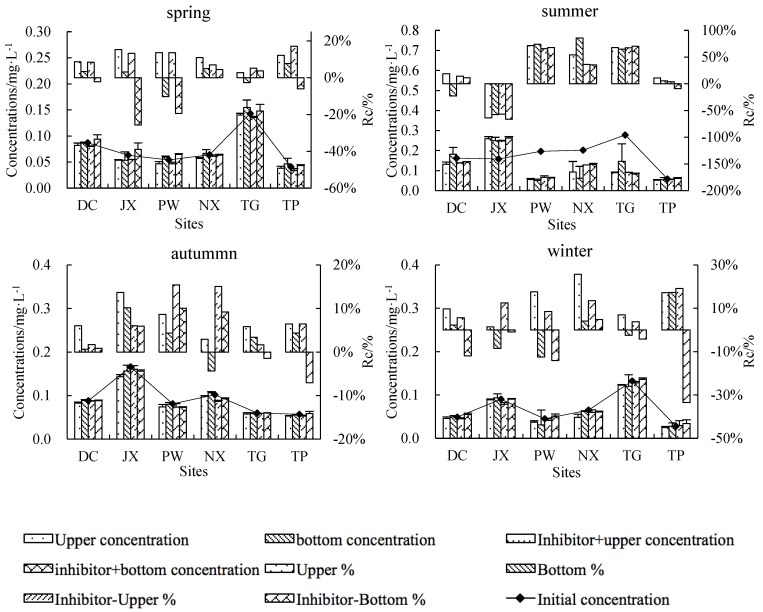
Initial NO_2_^−^-N concentrations (line), final upper- and bottom-layer NO_2_^−^-N concentrations (lower histogram) and percent change from initial NO_2_^−^-N concentrations (upper histogram). A negative percentage indicates an increase in concentrations and vice versa.

Being an intermediate of nitrification and denitrification, rates of change in NO_2_^−^-N concentrations are closely related to those of NH_4_^+^-N and NO_3_^−^-N. A decrease in NO_2_^−^-N concentrations was observed in 80% of samples, with the highest rates of change occurring in summer (−66.30–83.67%) (Figure 7).

Most data points illustrating the relationship between theoretical and observed bottom rates of change lie in quadrant I (19 points) and quadrant II (27 points), with only two points in quadrants III and IV (Figure 4). Points located in quadrant II are above the y = −x line, indicating a significant influence of biological activity across the four seasons.

### 3.2. Spatial and Seasonal Differences in N Reduction Rates

**Figure 8:**
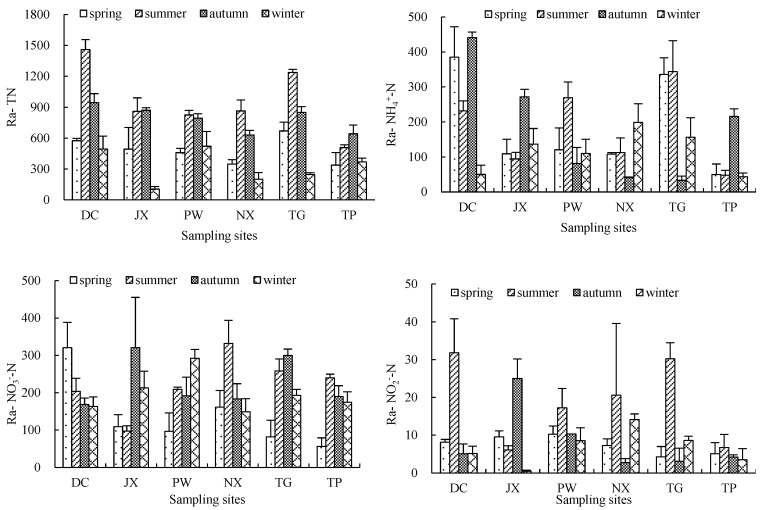
TN, NH_4_^+^-N, NO_3_^−^-N and NO_2_^−^-N reduction rates in the sampled rivers in different seasons (mg·(m^3^·d)^−1^).

Across the six rivers and four sampled seasons, TN, NH_4_^+^-N, NO_3_^−^-N and NO_2_^−^-N reduction rates ranged from 105.4 ± 25.3 to 1458.8 ± 98.4 mg·(m^3^·d)^−1^, 33.1 ± 12.3 to 440.9 ± 33.1 mg·(m^3^·d)^−1^, 56.3 ± 22.7 to 332.1 ± 61.9 mg·(m^3^·d)^−1^ and 0.4 ± 0.3 to 31.8 ± 9.0 mg·(m^3^·d)^−1^, respectively (Figure 8). TN reduction rates were significantly higher in summer and autumn than in other seasons (*p* < 0.05) at all sites but TP site. No significant differences of seasonal average TN reduction rates were observed across sampling sites except TP site (*p* > 0.05). Reduction rates of NH_4_^+^-N, NO_3_^−^-N and NO_2_^−^-N were higher in summer and autumn than in the remaining seasons at most sites, while in DC high spring NH_4_^+^-N and NO_3_^−^-N concentrations were observed. All six sites had significantly higher average NO_3_^−^-N reduction rates in autumn compared to spring, while NO_2_^−^-N reduction rates were highest in summer (*p* < 0.05).

We observed remarkable differences in NH_4_^+^-N, NO_3_^−^-N and NO_2_^−^-N reduction rates within the same season across rivers or within the same river across seasons. In spring, the reduction rates of NH_4_^+^-N in DC and TG and NO_3_^−^-N in DC were significantly higher than in other rivers (*p* < 0.05), while no significant difference was observed in NO_2_^−^-N reduction rates among rivers (*p* > 0.05). In summer, reduction rates of NH_4_^+^-N in TG, NO_3_^−^-N in NX and TG were significantly higher than in other rivers (*p* < 0.05), while NO_2_^−^-N reduction rates were significantly lower in JX and TP compared to others (*p* < 0.05). In autumn, DC had a significantly higher NH_4_^+^-N reduction rate than other rivers, while reduction rates of NO_3_^−^-N in JX, TG and NO_2_^−^-N in JX were significantly higher compared to other rivers (*p* < 0.05). In winter, the highest NH_4_^+^N, NO_3_^−^-N and NO_2_^−^-N reduction rates were observed in NX, PW and NX, respectively, while TN reduction rates were higher in DC than in other sites across the four seasons.

### 3.3. Microbial and Non-Microbial DIN Reduction Rates

**Figure 9:**
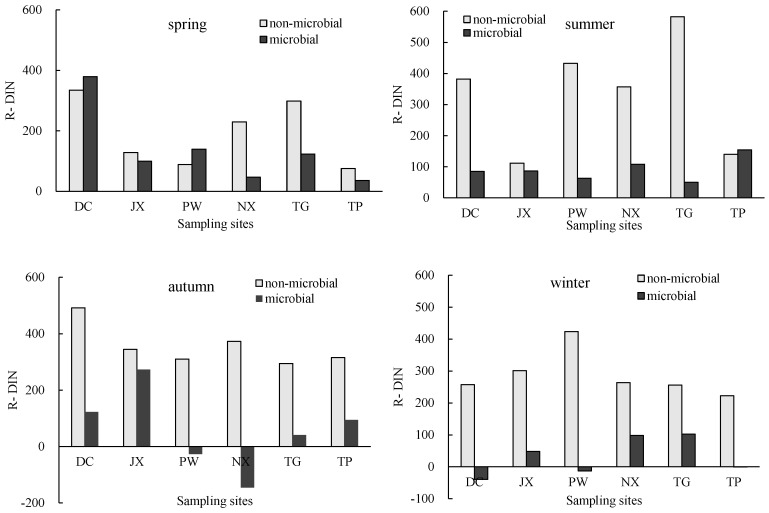
Microbial and non-microbial DIN (dissolved inorganic N) reduction rates at sampling sites in different seasons (mg·(m^3^·d)^−1^).

Different N reduce processes occurring in natural freshwater ecosystems, including microbial and non-microbial mechanisms, result in spatial and seasonal differences in DIN reduction rates. The relative contributions of microbial and non-microbial processes to DIN reduce differed among rivers and seasons (Figure 9). Across all sites and seasons, microbial DIN (MDIN) reduction rates ranged from −145.3 to 498.9 mg·(m^3^·d)^−1^, with an average of 96.0 ± 46.4 mg·(m^3^·d)^−1^, while non-microbial DIN (nMDIN) reduction rates ranged from 75.6 to 582.3 mg·(m^3^·d)^−1^, with an average of 288.1 ± 67.8 mg·(m^3^·d)^−1^. These results suggest that non-microbial processes were generally the primary DIN reduction mechanism.

In spring, MDIN and nMDIN reduction rates were higher in DC than in other sites. In summer, nMDIN reduction rates were generally higher than MDIN rates at all sites except TP, where nMDIN and MDIN rates were similar. In autumn and winter, nMDIN reduction rates were higher than MDIN rates at all sites. Negative MDIN reduction rates observed in PW, DC and NX might be due to sediment resuspension offsetting the effect of microbial reduce. The proportions of DIN reduction rates and N load attributable to microbial and non-microbial processes are shown in Table 2.

### 3.4. Factors Controlling Reduction Rates

We investigated environmental factors that may have affected N reduction rates observed in our in-situ incubation experiments. We found significant correlations between N reduction rates and initial N concentrations, DO, pH, suspended solids, water velocity and flow rate (Table 3). Specifically, N reduction rates were positively correlated with concentrations of N, pH and suspended solids and negatively correlated with DO, water velocity and flow rate. N reduce is a complex process influenced by a variety of environmental factors directly and indirectly, and the reduction of each N form considered in this study was also influenced by one or more factors.

We performed PCA analysis to explore correlations among variables and assess the relative importance of different factors. PCA results showed three significant components illustrating the effects of different environmental variables on N reduce in each season (Table 4). In spring, N concentrations and multiple environmental variables were the main factors affecting reduce processes, with 55.32% of the total variance explained. In summer, N concentrations and two water quality variables (DO and pH) explained 45.72% and 33.49% of the total variance, respectively, thus representing the main factors related to N reduce processes. In autumn, water velocity and flow and N concentrations were the most important factors, with 50.51% of the total variance explained, while N concentrations was the strongest driver in winter (53.45% of the total variance explained).

## 4. Discussion

### 4.1. Reduce Processes

Transformation and transport of N compounds in river water are complex processes involving physical, chemical and biological mechanisms. Basic N transformation processes include nitrification and denitrification [17], while vertical migration occurs through settlement of adsorbed compounds and desorption. Nitrification is typically considered an obligated aerobic and chemoautotrophic process [18], while denitrification is primarily a facultative heterotrophic process and occurs under low DO conditions [19,20]. We observed differences in TN, NH_4_^+^-N, NO_3_^-^-N and NO_2_^−^-N migration and transformation processes across different seasons (Figure 3 through Figure 7). The contribution of biochemical processes to TN reduction was weak in spring and autumn but strong in summer, while physical sedimentation dominated in winter (Figure 4). Biochemical transformation of NH_4_^+^-N, NO_3_^−^-N and NO_2_^−^-N occurred in our cultures and dominated in summer and autumn but became secondary in spring and winter. Higher temperatures in summer and autumn resulted in a strong contribution of microbial activities to the N cycle through nitrification and denitrification [21]. Nitrifying bacteria can convert NH_4_^+^ to NO_2_^−^ and then NO_3_^−^, as shown by our summer results in PW, where NH_4_^+^-N decreased by 96.25%, NO_2_^−^-N decreased by 69.93% and NO_3_^−^-N increased by 169.75%. The significantly greater decrease in NH_4_^+^-N in PW compared to other sites may be due to high concentrations of suspended solids (348 mg/L), which can promote nitrification in rivers [16,22]. Contrary to expectations, N concentrations decreased both in the upper and bottom culture water treated with a microbial inhibitor, with the decrease being particularly obvious for NH_4_^+^-N (Figure 4). This finding could be due to NH_4_^+^ volatilization resulting in a decrease in NH_4_^+^-N concentrations in the absence of microbial activity [23]. The 11 data points showing a decrease in NH_4_^+^-N mostly came from summer and autumn samples from NX, TG and TP, where NH_4_^+^ volatilization may be associated with extensive farmland areas around and upstream of the sampling sites (Figure 1), resulting in fertilizers being washed into rivers by rainfall. Suspended solids not only adsorb N compounds to their surface, but can also absorb N [24], which may explain the greater increase in bottom layer TN compared to the observed decrease in the corresponding upper layer (Figure 4, upper left panel). Our in-situ experiment allowed us to investigate the main processes contributing to transport and transformation of different N forms across different rivers and seasons. However, the current experimental design does not allow us to quantify the relative contribution of each process to different N forms.

### 4.2. Analysis of Spatio-Temporal Differences

Processes contributing to observed decreases in DIN (NH_4_^+^, NO_3_^−^ and NO_2_^−^) are complex and include microbial and non-microbial mechanisms. Nitrification, the production of NO_3_^−^ from NH_4_^+^, and denitrification of NO_3_^−^ to gaseous N are the main microbial reduce processes. DIN can also be adsorbed by suspended solids and leave the water through deposition. These processes are affected by different river conditions.

According to the results of our in-situ experiment, reduction rates of NH_4_^+^, NO_3_^−^ and NO_2_^−^ showed significant seasonal and spatial heterogeneity. In the six study rivers, NH_4_^+^-N, NO_3_^−^-N and NO_2_^−^-N reduction rates ranged from 33.1 ± 12.3 to 440.9 ± 33.1 mg·(m^3^·d)^−1^, 56.3 ± 22.7 to 332.1 ± 61.9 mg·(m^3^·d)^−1^ and 0.4 ± 0.3 to 31.8 ± 9.0 mg·(m^3^·d)^−1^, respectively, across four seasons. Reduction rates of NH_4_^+^-N and NO_2_^−^-N were positively correlated with NH_4_^+^-N and NO_2_^−^-N initial concentrations, while a similar correlation was not observed between NO_3_^−^-N reduction rates and initial NO_3_^−^ concentrations (Table 3). In Lake Taihu’s Zhushan Bay, a maximum NH_3_ oxidation rate of 224 mg (m^3^·d)^−1^ was measured in relation to an NH_3_ concentrations of 0.85 mg/L [25], which is lower than the maximum values observed in this study. The NH_4_^+^-N reduction rates measured in this study including microbial (nitrification and anaerobic NH_3_ oxidation) and non-microbial processes showed that 39.62% of the reduce was attributable to microbial activity, indicating that our results are plausible. Several studies have reported nitrification rates in different aquatic systems. For example, nitrification rates of 249 mg N (m^2^·d)^−1^ and 370 mg N (m^2^·d)^−1^ were measured in sediments of a Lake Superior estuary and Onondaga Lake, respectively [12,26]. Both studies differed from our work and focused on sediment nitrification rates, though nitrification in the water column is often not negligible. The activity of nitrifying bacteria is regulated by NH_3_-N, with NH_3_ oxidizing bacteria increasing significantly at high NH_3_-N levels, thereby promoting NH_3_ oxidation [27]. At the same time, suspended solids can promote nitrification by providing microbial adhesion sites [16] and can also contribute to removing NH_4_^+^ from the water column through bottom deposition. NH_4_^+^-N reduction rates were negatively correlated with water velocity and flow rate. Previous studies have shown that heavy navigation traffic and wider river can accelerate pollutant degradation and dilution, thereby decreasing pollutant concentrations [9,28]. Water turbulence also affects suspended solid settlement rates. In this study, NO_3_^−^-N reduction rates were not correlated with NO_3_^−^-N concentrations, but a positive correlation was observed between microbial NO_3_^−^-N reduction rates and suspended solid content. In Poyang Lake, denitrification rates ranging from 0.25 ± 0.18 to 0.63 ± 0.24 μmol N·L^−1^, corresponding to ca. 3.5 to 8.8 mg N (m^3^·d)^−1^, suggested the occurrence of denitrifying processes on the surface of suspended solids [15]. Previous studies have reported a correlation between denitrification rates and NO_3_^−^ content. In this study, NO_3_^−^-N reduction rates were negatively correlated with DO concentrations, similar to the findings of Wang (2015), who reported a significant correlation between N_2_O production rates and DO. Denitrification can be affected by suspended solids, whose surface can provide microscopic interstices characterized by anaerobic conditions for denitrification to occur. In addition, NO_3_^−^-N adsorbed on the surface of suspended solids can be removed from the water column through deposition. NO_2_^−^-N reduction rates showed a significant positive correlation with initial NO_2_^−^ concentrations and a negative correlation with DO. As the substrate for the second part of the nitrification process, NO_2_^−^ concentrations can directly affect nitrification rates. NO_2_^−^ concentration is generally the primary factor affecting nitrification rates under limiting NO_2_^−^ levels, with other environmental factors becoming significant when NO_2_^−^ is not limiting.

### 4.3. DIN Reduce Processes

In this study, the fraction of DIN reduction rates associated with non-microbial processes was higher than that accounted for by microbial activities, suggesting that adsorption and sedimentation had a stronger effect than biotransformation on DIN reduction rates. However, sedimentation of suspended matter is expected to be less prominent in a natural river setting than what we observed in our study. Because our experiment was conducted in a glass culture flask and no external water was supplied continuously, we may have overestimated the relative contribution of non-microbial processes on DIN reduce. Nevertheless, our results on the main DIN transformation processes and the overall effects of non-biological and microbial processes on spatio-temporal differences are realistic, because we ensured identical in-situ experimental conditions among seasons and sampling sites. Spring and summer DIN reduction rates in the absence of microbial activity varied greatly because of strong differences in flow velocity among sampling sites (Table 1), as indicated by the observed relationship between non-microbial DIN reduction rates, water velocity and pH (Table 3) [29]. Coupled nitrification-denitrification is the main biological process contributing to DIN reduce [30,31,32]. We found no correlation between DIN reduction rates and concentrations of different N forms, suggesting that N compounds were present in sufficient concentrations and did not represent a limiting factor [13]. We found a significant positive correlation between microbial DIN reduction rates and suspended solids, indicating that the presence of suspended solids can accelerate nitrification-denitrification processes and promote DIN reduce [15,22,33].

We estimated that N reduce by microbial activities accounted for 7.9–61.2% of the DIN load (average: 29.7%) and 0–6.4% of the N load (average: 2.2%). Our results are consistent with previous studies, which estimated that the amount of N removed by denitrification accounted for 4.6% and 6.3% of the N load in the Mara River Basin and in Poyang Lake, respectively [15,34].

## 5. Conclusions

In natural conditions, N transformation and transport mechanisms mediated by biological and non-biological processes can reduce the N content in river water. In order to determine the differences in reduce processes and rate of different N forms and assessed the contribution of biological processes to dissolved inorganic N (DIN) reduce and the effects of different environmental factors on N reduce processes, we designed an experimental apparatus to conduct in-situ culture experiments in six rivers in the Taihu Lake Basin. We found that reduction rates of different N forms were higher in summer and autumn than spring and winter, with biological and non-biological processes playing a stronger role in summer and winter, respectively. Biological and non-biological DIN reduction rates differed spatially and temporally. Although non-biological processes played a major role in N reduce, biological processes are the main mechanism that can permanently remove N from the water column. Various factors affected the observed spatio-temporal differences in N reduction rates. The main influencing factors were the concentrations of different N forms and multiple environmental factors in spring, N concentrations, DO and pH in summer, N concentrations and water velocity in autumn and N concentrations in winter. Although our results may overestimate the effects of non-microbial processes in removing N forms due to the design characteristics of our experimental apparatus, the observed temporal and spatial patterns were not affected and can be considered realistic.

## Figures and Tables

**Table 1:** Physico-chemical parameters measured at each sampling site in spring, summer, autumn and winter.

**Sites**	**DO (mg·L^−1^)**				**pH**				**Velocity (m·s^−1^)**			
	**Spring**	**Summer**	**Autumn**	**Winter**	**Spring**	**Summer**	**Autumn**	**Winter**	**Spring**	**Summer**	**Autumn**	**Winter**
DC	7.53	2.60	4.00	6.30	7.70	7.95	8.34	7.54	0.01	0.01	0.04	0.18
JX	8.21	2.65	4.11	5.03	7.83	7.67	8.26	7.71	0.06	0.22	0.15	0.07
PW	10.53	4.73	3.34	5.91	7.31	7.99	8.04	7.67	0.05	0.11	0.17	0.03
NX	6.70	5.72	4.53	5.71	7.66	8.23	7.77	8.25	0.35	0.08	0.13	0.05
TG	7.19	8.74	4.66	6.40	8.01	8.62	7.88	7.32	0.16	0.05	0.13	0.11
TP	10.93	5.17	3.62	6.36	7.24	8.03	7.98	7.64	0.30	0.50	0.28	0.42
**Sites**	**Water Flow (m^3^·s^−1^)**				**SS (mg·L^−1^)**				**River Width (m)**		**River Cross-Sectional Area (m^2^)**	
	**Spring**	**Summer**	**Autumn**	**Winter**	**Spring**	**Summer**	**Autumn**	**Winter**				
DC	0.73	1.56	3.36	15.39	198.50	52.13	89.50	19.90	45.96		109.70	
JX	5.26	31.07	22.20	6.72	40.50	35.37	150.00	5.65	69.13		141.97	
PW	15.68	45.13	58.04	9.47	19.50	348.00	65.50	12.15	95.35		393.78	
NX	5.35	11.74	15.95	5.56	25.50	5.79	85.50	7.00	64.06		145.93	
TG	3.59	3.37	7.53	5.02	23.50	9.75	54.00	5.95	29.60		67.87	
TP	239.69	464.01	250.03	331.89	25.00	22.20	52.00	6.35	159.68		921.654	

DO: Dissolved oxygen; SS: Suspended solids.

**Table 2:** Proportion of DIN (dissolved inorganic N) reduction rates and N load attributable to microbial and non-microbial processes (%).

Sites		Spring		Summer		Autumn		Winter	
		DIN	N	DIN	N	DIN	N	DIN	N
DC	non-microbial	46.9	5.7	81.8	7.0	80.0	5.8	-	6.2
JX		56.4	2.4	56.4	2.2	55.8	6.4	86.2	5.9
PW		38.8	-	87.2	7.7	-	8.1	-	7.7
NX		83.1	4.4	76.7	9.6	-	10.0	72.9	5.9
TG		70.8	4.0	92.1	15.7	87.6	7.1	71.5	3.6
TP		67.8	-	47.5	4.8	77.0	11.1	-	7.5
DC	microbial	53.1	6.4	18.2	1.6	20.0	1.5	-	-
JX		43.6	1.9	43.6	1.7	44.2	5.1	13.8	0.9
PW		61.2	2.3	12.8	1.1	-	-	-	-
NX		16.9	0.9	23.3	2.9	-	-	27.1	2.2
TG		29.2	1.7	7.9	1.3	12.4	1.0	28.5	1.4
TP		32.2	0.9	52.5	5.3	23.0	3.3	-	0.0

DIN% and N% represent the proportion of DIN reduction rates and N load attributable to microbial and non-microbial processes. Dashes indicate cases where percentages <0% or >100% were obtained.

**Table 3:** Correlation coefficients between N reduction rates and environmental factors.

Reduction Rates		NH_4_^+^-N	NO_3_^−^-N	NO_2_^−^-N	DO	Suspended Solids	pH	Velocity	Water Flow
TN	control	-	-	0.557(+)	0.272(−)	-	0.304(+)	0.163(−)	-
	non-microbial	0.243(+)	-	0.255(+)	-	-	-	0.261(−)	0.242(−)
	microbial	-	-	0.291(+)	0.329(−)	-	-	-	-
NH_4_^+^-N	control	0.455(+)	-	0.237(+)	-	0.142(+)	0.249(+)	0.243(−)	0.242(−)
	non-microbial	0.296(+)	-	0.427(+)	-	-	0.327(+)	0.190(−)	0.138(−)
	microbial	0.226(+)	-	-	-	0.460(+)	-	-	-
NO_3_^−^-N	control	-	-	-	0.142(−)	-	-	-	-
	non-microbial	-	-	-	0.287(−)	-	-	-	-
	microbial		-	-	-	0.206(+)		-	-
NO_2_^−^-N	control	-	-	0.502(+)	-	-	0.249(+)	-	-
	non-microbial	-	-	0.451(+)	-	-	-	-	-
	microbial	-	-	0.218(+)	0.128(−)	-	-	-	-
DIN	non-microbial	-	-	0.279(+)	0.170(−)	-	0.418(+)	0.198(−)	0.134(−)
	microbial	-	-	-	-	0.416(+)	-	-	-

Values represent the fitting coefficient for correlation; “-” represent no correlation; (+), (−) represent the positive or negative correlation.

**Table 4:** PCA (principal component analysis) loadings, eigenvalues and percentages of variance explained for environmental parameters measured at all sampling sites across four seasons.

Parameters	Spring			Summer			Autumn			Winter		
	PC1	PC2	PC3	PC1	PC2	PC3	PC1	PC2	PC3	PC1	PC2	PC3
DO	**−0.766**	0.033	−0.425	−0.219	**0.834**	0.487	0.281	0.330	**−0.794**	−0.242	**0.813**	0.301
pH	**0.866**	0.022	0.274	−0.110	**0.911**	0.367	0.680	−0.391	0.582	−0.277	**−0.818**	−0.242
velocity	−0.403	0.452	**0.756**	−0.734	−0.541	0.356	**−0.790**	0.347	0.473	−0.738	0.597	−0.247
flow	**−0.824**	−0.083	0.341	−0.718	−0.452	0.521	**−0.762**	−0.107	0.543	**−0.765**	0.449	−0.446
SS	0.279	**−0.934**	−0.045	0.734	−0.329	0.516	**0.804**	0.457	0.354	−0.246	0.000	**0.964**
TN	**0.923**	0.165	−0.166	**0.896**	−0.337	−0.263	**0.831**	−0.548	0.001	**0.969**	0.181	0.055
NH_4_^+^	**0.829**	−0.251	0.249	**0.898**	−0.274	0.328	0.545	**−0.815**	0.028	**0.865**	0.435	−0.140
NO_3_^−^	0.623	0.647	−0.412	**−0.765**	0.100	−0.523	**0.794**	0.597	0.080	**0.986**	0.024	−0.012
NO_2_^−^	**0.892**	0.036	0.268	0.516	**0.827**	−0.108	0.732	0.578	0.342	**0.909**	0.195	−0.221
Eigenvalue	4.979	1.595	1.278	4.115	3.014	1.499	4.546	2.256	1.738	4.811	2.147	1.409
% of variance	55.320	17.719	14.197	45.720	33.491	16.658	50.508	25.062	19.312	53.452	23.856	15.659

Absolute loading values > 0.750 are indicated in bold.
